# WCS-YOLOv8s: an improved YOLOv8s model for target identification and localization throughout the strawberry growth process

**DOI:** 10.3389/fpls.2025.1579335

**Published:** 2025-07-11

**Authors:** Sheng Gao, Gongpei Cui, Qiaohua Wang

**Affiliations:** ^1^ School of Information and Control Engineering, Qingdao University of Technology, Qingdao, China; ^2^ College of Biological Engineering, Qingdao University of Science and Technology, Qingdao, China; ^3^ College of Mechanical and Electrical Engineering, Henan Agricultural University, Zhengzhou, China; ^4^ College of Engineering, Huazhong Agricultural University, Wuhan, China

**Keywords:** strawberry, deep learning, target recognition and localization, WCS-YOLOv8s model, binocular vision

## Abstract

**Introduction:**

To enhance the quality and yield of strawberries, it is essential to effectively supervise the entire growing process. Currently, the monitoring of strawberry growth primarily relies on manual identification and positioning methods. This approach presents several challenges, including low efficiency, high labor intensity, time consumption, elevated costs, and a lack of standardized monitoring protocols. On the basis of this, there was an urgent need in the market to automate the whole process of target recognition and localization in strawberry growing.

**Methods:**

Aiming at the above problems, we innovatively constructed a model for target recognition and localization of strawberries based on the YOLOv8s benchmark model, named the WCS-YOLOv8s model. In this paper, the whole growth process of the strawberry was divided into four stages, namely, the bud, flower, fruit under-ripening, and fruit ripening stages, and a total of 1,957 images of these four stages were captured with a binocular depth camera. Using the constructed WCS-YOLOv8s model to process the images, the target recognition and localization of the whole growth process of the strawberry were accomplished. This model proposes a data enhancement strategy based on the Warmup learning rate to stabilize the initial training process. The self- developed SE-MSDWA module is integrated into the backbone network to improve the model’s feature extraction capability while suppressing redundant information, thereby achieving efficient feature extraction. Additionally, the neck network is enhanced by incorporating the CGFM module, which employs a multi-head self-attention mechanism to fuse diverse feature information and improve the network’s feature fusion performance.

**Results and discussion:**

The model’s Precision (P), Recall (R), HYPERLINK "mailto:mAP@0.5" mAP@0.5, and mAP@0.5:0.95 of detection were 83.4%, 86.7%, 87.53%, and 60.48%, respectively, and the detection speed was 45.9 FPS(21.8 ms/per image, which significantly improved on the detection accuracy and generalization ability of with the YOLOv8s benchmark model. This model can meet the demand for online real-time target identification and localization of strawberries and provide a new detection method for the automated monitoring and management of the whole growth process of strawberries.

## 1 Introduction

Strawberry is a common fruit, with a sweet taste that is loved by people, and is known as the ‘Queen of Fruits’. Strawberries are rich in free sugars, organic acids, and other important ingredients that have health benefits such as protecting one’s eyesight and promoting digestion and have anti-inflammatory properties (Ikegaya, 2024; Newerli-Guz et al., 2023; Gasparrini et al., 2017; Afrin et al., 2016). At present, there is an increasing demand for strawberry fruits, however, due to the complexity of fruit identification and localization during strawberry growth, the level of intelligent and mechanized strawberry fruit picking is still very low, and relying on manual completion is increasingly failing to satisfy the market's demand for strawberries (Zhao et al., 2022; Ibba et al. 2021).

Flower and fruit thinning is an important part of orchard management, directly affecting fruit yield and quality and preventing early plant failure. During the strawberry growth process, the best times for flower thinning and fruit thinning are the bud and flower stages. Research has shown that rational flower and fruit thinning can remove deformed, diseased, and excessive fruits, helping to regulate the plant’s nutrient supply to the fruits, improving fruit quality, and increasing the yield by 20%–30% (Domingos et al., 2016; Yu et al., 2023). The key to automating flower thinning, fruit thinning, and picking is to achieve target identification and localization of strawberries (Castle et al., 2019). Most of the current research focused on the detection of ripe strawberries, while less research had been conducted on the strawberry bud and flower stages. The research in this paper included the bud stage and blossom stage during strawberry growth, which could provide technical support for the realization of automated flower thinning, fruit thinning, and picking of strawberries.

Computer vision has been widely applied in agriculture, food, transportation, and other fields (Zhao et al., 2019; Zou et al., 2023; Yang et al., 2023; Babu et al., 2023; Singh et al., 2023). The use of computer vision technology to identify strawberries has broad application potential and provides theoretical support for robot picking and automated orchard management in strawberry production. Currently, strawberry picking mainly relies on manual labor, where workers rely on their own experience to identify and pick ripe strawberries. However, due to inconsistent evaluation standards and the diversity of strawberry varieties, the optimal picking period is often missed (Ge et al., 2023). Traditional methods for detecting flowers and fruits mainly involve machine vision techniques that autonomously extract features such as shape, texture, and size based on human experience (Rizon et al., 2015). For example, Lin et al. proposed a support vector machine (SVM) model for identifying citrus and tomatoes based on color and contour features (Lin et al., 2020). Guru et al. achieved flower classification by using threshold segmentation methods and feature extraction on flower images (Guru et al., 2011). Xu et al. used hue, saturation, value (HSV) to detect strawberries. color information to detect strawberry regions and combined this information with an SVM classifier (Xu et al., 2013). Although these methods offer some solutions, the manual extraction of features based on personal experience makes it difficult to extract deep feature information from images, resulting in lower robustness and recognition accuracy of models built using traditional machine vision techniques (Ma et al., 2021). In contrast, deep learning technology, by extracting deeper features from image data, has improved the accuracy and speed of object detection in complex environments (Wang et al., 2022). Deep learning technology has been widely applied in the detection research on strawberry, apple, and other fruit flowers for maturity and yield (Guo et al., 2022; Ismail and Malik, 2022; Wang et al., 2021; Wang and He, 2021). Font et al. developed a computer vision system based on color and specular reflection patterns for the rapid and accurate estimation of apple orchard yields. However, this system had the drawback of relying on artificial lighting at night to reduce the influence of natural light (Font et al., 2014). Lin et al. established a strawberry flower detection algorithm based on Faster R-CNN, achieving the detection of strawberry flowers in outdoor environments with overlapping flowers and complex backgrounds (Lin et al., 2020). Zhang et al. reduced the number of convolutional layers and CBL modules in the CSPNet backbone and established a real-time strawberry monitoring algorithm based on YOLOv4 Tiny, achieving rapid and real-time detection of strawberries (Zhang et al., 2022). Binocular cameras have gradually been applied in the research of target recognition and positioning. Qi et al. established a TCYOLO algorithm with CSPDenseNet and CSPResNeXt as the dominant networks, achieving accurate detection of chrysanthemum flowers (Qi et al., 2022). Hu et al. used a ZED stereo camera to perform three-dimensional positioning of strawberries. The strawberry detection and positioning method proposed in the study can effectively provide the precise location of mature strawberries for picking robots (Hu et al., 2022). Fu et al. improved the YOLOv3-tiny model and developed an algorithm for the automatic, rapid, and accurate detection of kiwifruit in orchards. The experimental results showed that the improved model is small and efficient, with high detection accuracy (Fu et al., 2021). Bai et al. built a YOLO real-time recognition algorithm to achieve accurate flower and fruit recognition of strawberry seedlings in a greenhouse (Bai et al., 2024). However, there has been no research on the use of binocular positioning cameras for target recognition and positioning of the entire growth process of strawberries (bud, flower, unripe, and ripe stages) nor has there been any research addressing the practical needs of orchards for automated thinning of flowers and fruits and the detection and positioning of mature strawberries. Orchards urgently need to achieve automated management of the entire growth process of strawberries.

In this paper, a new model of strawberry target identification and localization based on the YOLOv8s model, named the WCS-YOLOv8s model, is innovatively proposed for the four stages of the strawberry growth process (bud, flower, fruit under-ripening, and fruit ripening stages) that provides supervision of the whole growth process of strawberries. The model provided a reliable new method for target identification and localization for the automated supervision of the whole strawberry growth process, leading to fruit picking and quality improvements. The improvement and innovation points of this paper include:

1. A data enhancement strategy based on the Warmup learning rate is proposed in this paper, which could provide a stable convergence direction for the model and avoid oscillations at the early stage of training.
2. The model introduced the Context Guide Fusion Module (CGFM), which used the multi-head self-attention mechanism to fuse different information and improve the recognition accuracy in complex scenes.
3. The model proposed the Squeeze-and-Excitation-Enhanced Multi-Scale Depthwise Attention (SE-MSDWA) module, which combined multi-scale convolution and SEAttention to enhance the feature extraction efficiency of the samples and significantly improved the detection effect of the model in complex scenes.

## 2 Materials and methods

### 2.1 Sample collection and dataset construction

The samples were collected from March to May 2024. The collection site was Hongshiyi strawberry planting orchard in Shandong Province (121.49°E, 36.77°N). A total of 1,957 sample images (image size of 640 × 640 pixels) were collected. The sample varieties included ‘Sweet Treasure’, ‘Red Face’, ‘Fengxiang’, ‘Miaoxiang’, and ‘Zhangji’.

The sample collection tools were a laptop CPU: Intel(R) Xeon(R) E5–2673 v4; GPU: NVIDIA 3090; and an Intel^®^ RealSense D435i binocular depth camera (Intel^®^, United States of America; depth resolution and frame rate are 1280×720 and 90 FPS (maximum), respectively; binocular detection range of 0.105–10 m). The image dataset was acquired utilizing the above laptop, instrument, and the setting parameters. The sample collection method involved collecting sample images using a laptop computer connected to an Intel^®^ D435i camera (shooting distance of 0.3–0.8 m) from 07:00 to 19:00 every day in 10 sessions. Sample data were collected from different growing sheds to eliminate data bias due to geographical location and variety. Images contained bright and shady light and complex environments and backgrounds. The dataset was randomly divided into three subsets: training set, validation set, and testing set, with a ratio of 7:2:1.

**Figure 1:**
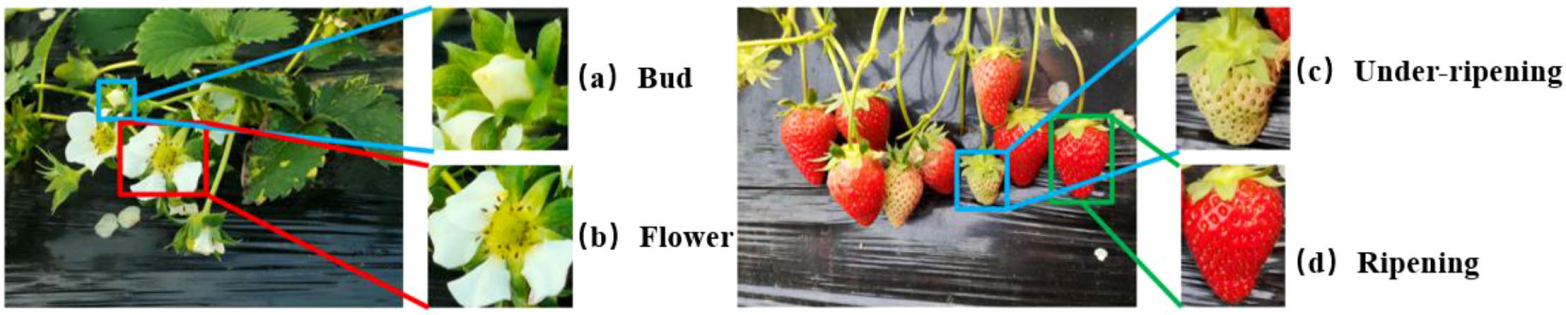
Presentation and labeling of selected datasets. **(a–d)** are the four growth states of strawberry bud, flower, under-ripening, ripening respectively.

This paper classified the samples into four stages according to the fruit growers’ planting experience, namely, the bud, flower, fruit under-ripening, and fruit ripening stages, and collected image data for these four stages. The identification criteria for immature strawberries were that the color of the fruit was light red or green covering a large area, the fruit was not full, and the size was slightly small. The identification criteria for mature strawberries were that the color of the fruit was bright red and the fruit was large and full. Some samples are shown in **Figure 1**. In **Figures 1a–d** are strawberry samples in the bud, flower, fruit under-ripening, and fruit ripening stages, respectively.

### 2.2 WSC-YOLOv8s model construction

#### 2.2.1 Overall structure of the WSC-YOLOv8s model

YOLOv8 is a powerful real-time object detection algorithm that uses an end-to-end architecture to achieve regression and prediction of a target’s category and location using feature extraction and fusion of input images through convolutional neural networks. The YOLOv8 framework is divided into four main components: input layer, backbone network, neck network, and prediction layer (Simanjuntak et al., 2024).

**Figure 2:**
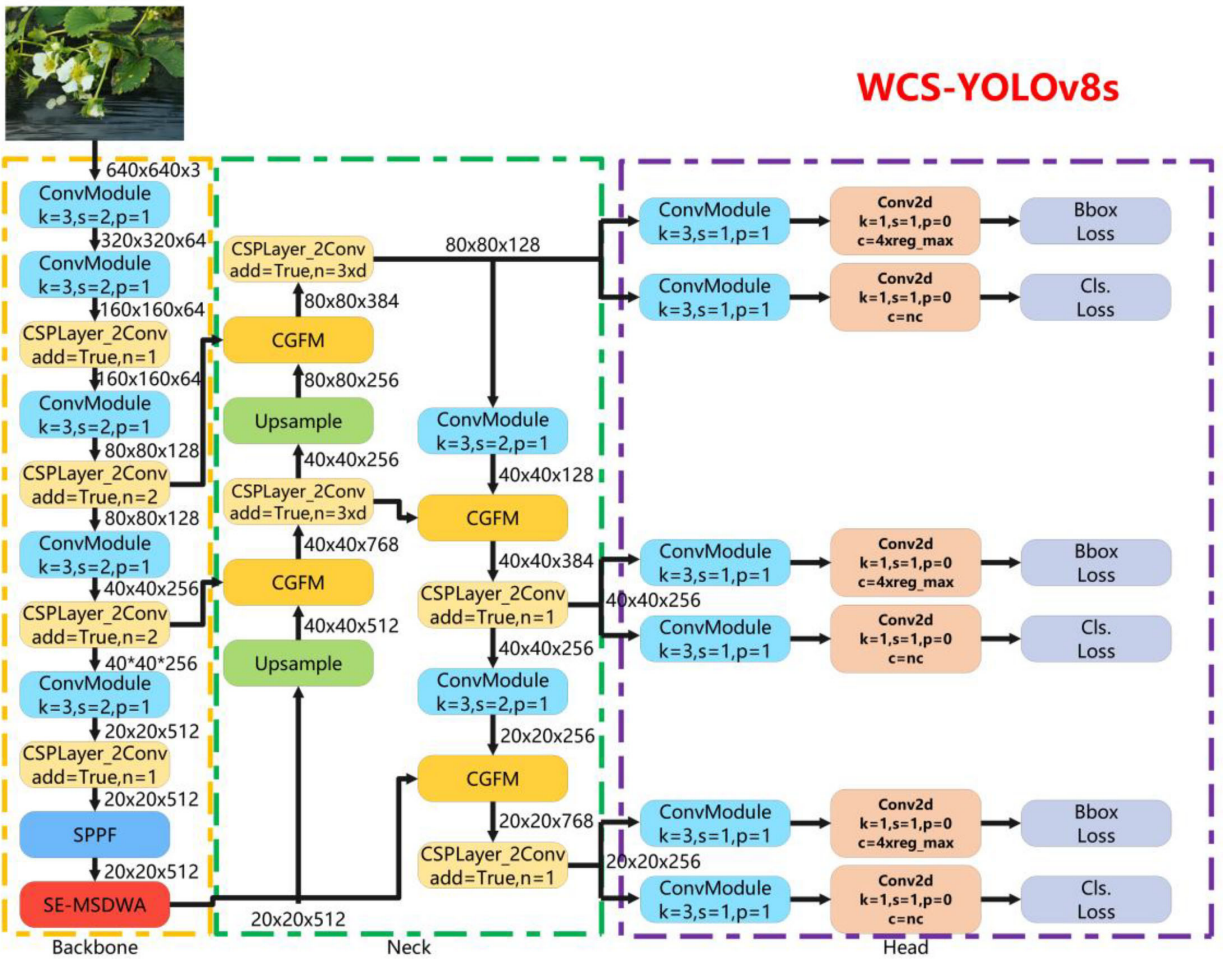
Network framework diagram for the WCS-YOLOv8s model.

In this paper, improvements were made to YOLOv8s. First, Warmup data augmentation was used, i.e., the original data augmentation strategy was changed to gradually increase the probability of data augmentation occurring as the epoch changes. Second, the self-developed SE-MSDWA module was applied at the end of the backbone network to achieve efficient feature extraction, ensuring the model focused on the region of interest. Finally, the neck network was improved by using the CGFM module to enhance the feature fusion performance of the network. Based on the above, this paper constructed the WCS-YOLOv8s model for target identification and localization during the whole strawberry growth process, and the network framework of the constructed model is shown in **Figure 2**.

#### 2.2.2 Data enhancement with Warmup

Warmup was first mentioned in ResNet as a way to optimize for learning rate (Nakamura et al., 2021). The method of using Warmup to warm up the learning rate causes the model to gradually stabilize at a smaller learning rate during the first few epochs of training, and when the model is stabilized, it can then be trained using the pre-set learning rate, which speeds up the convergence of the model and improves the model effect. The initial use of lower probability data transformation helps the model to learn the relationship between samples, improving the adaptability to different data distributions, causing the model to enter the training process smoothly, and avoiding falling into the local optimal solution. Gradually increasing the probability of sample transformation with the training process further enhances the generalization ability of the model and improves the robustness. Through Warmup data enhancement, the model learns and generalizes effectively.

In this paper, data augmentation was performed at the beginning of training using smaller probabilities. When the training proceeded to 1/5 of the total number of rounds, all data enhancements were performed as default in YOLOv8. Equation 1 for the variation of data enhancement probability is shown below:


(1) $$\begin{array}{l} N=\frac{Current\_epoch*5}{Total\_epochs}\\ P=\left\{ \begin{array}{l} 1\;,\text{ if }N>=1\\ N,\;otherwise \end{array}\right. \end{array}$$

Where current_epoch is the current round number in the training process and total_epochs is the total number of rounds in the training process.

#### 2.2.3 SE-MSDWA module in the model

**Figure 3:**
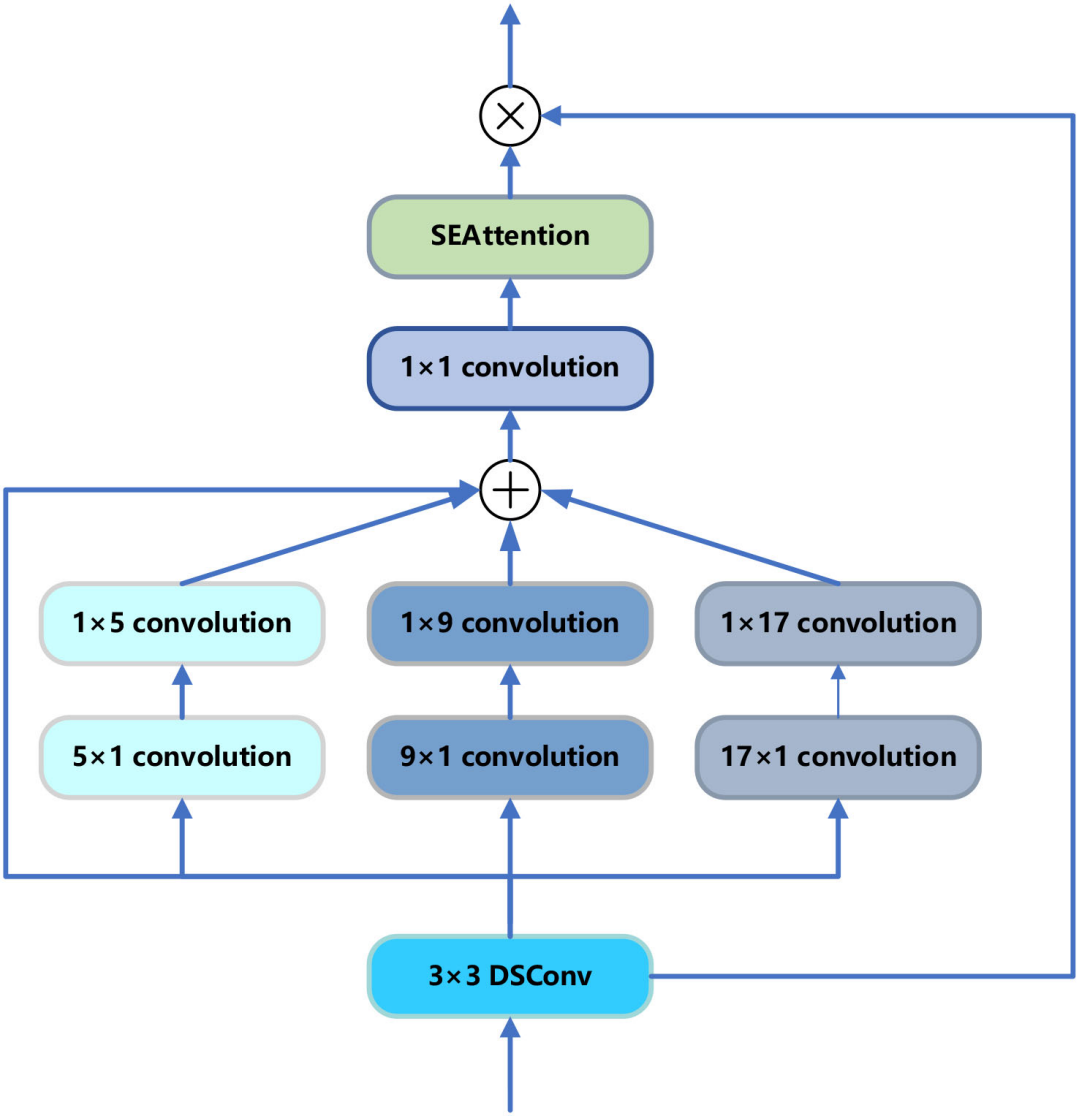
Structure of the SE-MSDWA module.

The SE-MSDWA module aimed to enhance the feature extraction capability and overall performance of convolutional neural networks by combining depth-separable convolution, multi-scale convolution, and SE blocks. The SE-MSDWA module first performed convolutional operations on each channel independently through deep convolution to extract important spatial information and then interacted with the information between channels through point-by-point convolution. Second, the module used three sets of convolution kernels with different scales to perform multi-scale convolution processing: the ranges of convolution kernels captured by [(1, 5) and (5, 1)], [(1, 9) and (9, 1)], and [(1, 17) and (17, 1)] were smaller, medium, and larger features, respectively. After these convolutional processes, the feature maps were fused with multi-scale information through an additional convolutional layer and finally entered into the SE module. The SE module first performed adaptive average pooling to reduce the feature map of each channel to 1x1, computed the weights of each channel through two fully connected layers and activation functions, and reapplied these weights to the original feature map, as shown in **Figure 3**.

The SE-MSDWA module effectively solved the deficiencies of traditional convolutional layers in capturing multi-scale features and handling feature redundancy. The module significantly enhanced the feature representation capability of the network by dynamically adjusting the channel weights, thus improving the performance of the model in various computer vision tasks. The module enhanced the network’s adaptability in different scenes and tasks through multi-scale feature extraction and attention mechanisms.

#### 2.2.4 CGFM modules in the model

**Figure 4:**
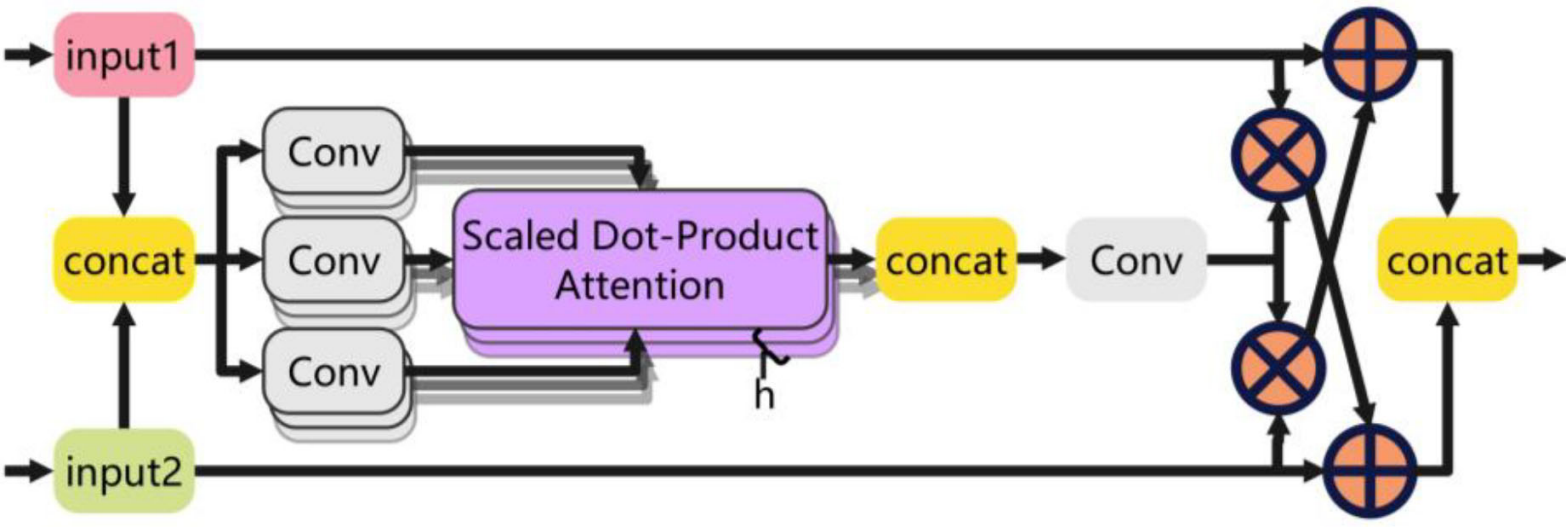
CGFM of the model.

Concat has its limitations and drawbacks in deep learning and cannot give full play to the complementary effects of different features. On the basis of this, this paper proposed the CGFM, a feature fusion structure based on a self-attention mechanism to improve the performance and efficiency of the model. The CGFM is an innovative feature fusion module designed to improve the Feature Pyramid Network (FPN) in YOLOv8s. Through the multi-head self-attention mechanism, the CGFM module adjusts the number of channels by splicing two different feature maps, input1 and input2, in the channel dimension, which are processed by the multi-head self-attention mechanism of convolution after splitting, and then the number of channels is adjusted by convolution. Second, the split data are multiplied by elements with the two inputs respectively and added to the other input to get the blended features. Finally, the two are spliced to achieve feature fusion and cross-interaction, which improves the feature fusion capability of the neck network. The CGFM enhances the important features using the multi-head self-attention mechanism, which effectively suppresses the unimportant features and improves the discriminative power and visual performance of the fused features through detail enhancement. A detailed structure of the CGFM is shown in **Figure 4**.

## 3 Results and discussion

### 3.1 Experimental platform

Model training and evaluation were performed using the following computers and operating systems. The experimental platform configuration parameters were as follows: CPU: Intel(R) Xeon(R) E5–2673 v4, GPU: NVIDIA 3090, OS: Ubuntu. The programming language used for the experiment was Python 3.8.19. To enhance the efficiency of the model training, a CUDA 11.6 accelerator was introduced. In the experiment, the resolution of the input image was 640 × 640 pixels and 32 samples were processed in batches for each training module. The Adam optimizer was employed with an initial learning rate of 0.01. The learning rate was automatically optimized by the cosine annealing learning rate decay algorithm, and after 100 training cycles, the best model weight parameter file was saved and used for model evaluation (Yang et al., 2022).

### 3.2 Evaluation indicators

To comprehensively evaluate the performance of the constructed model, this paper introduced multiple evaluation metrics to quantify both the model’s effectiveness and its resource consumption in practical applications. The evaluation metrics employed in this paper encompass precision (P), recall (R), average precision (AP), mean average precision (mAP), model parameters, floating point operations per second (FLOPs), and detection frame rate (FPS). With Intersection over Union (IoU), it is possible to visualize the degree of match between the target detection results and the real situation.

P measures the proportion of correctly detected targets to all detected targets by the model and reflects the accuracy of the model in identifying positive class objects. Equation 2 is calculated as follows:


(2) $$P=\frac{TP}{TP+FP}$$

Where true positive (TP) denotes the number of positive samples recognized, false positive (FP) denotes the number of negative samples misreported, and FN denotes the number of positive samples missed. N denotes the number of sample categories.

R represents the proportion of targets correctly detected by the model to all actual positive class targets, revealing the model’s ability to cover positive class samples. Equation 3 is calculated as follows:


(3) $$R=\frac{TP}{TP+FN}$$

AP is the average of precision rates at different levels of recall and provides an assessment of performance for a single category. The calculation of Equation 4 is as follows:


(4) $$AP=\int_{0}^{1}P(R)dR$$

mAP assesses the performance of multi-category object detection by calculating the average of the AP values across all categories. This metric effectively evaluates the accuracy of the model in detection tasks. mAP@0.5 and mAP@0.5:0.95 are two commonly used metrics for evaluating the mAP and, thus, they were selected for the evaluation in this paper. mAP@0.5 was compared with mAP@0.5:0.95. mAP@0.5:0.95 indicates the average mAP calculated under multiple IoU thresholds (from 0.5 to 0.95 in steps of 0.05). This means that the model’s performance under multiple different IoU thresholds was taken into account, providing a more comprehensive evaluation. In this paper, the whole growth process of the strawberry was divided into four stages, and the four stages correspond to four categories. Target detection and localization of the four categories were performed. In this paper, mAP was employed as a crucial evaluation metric, with mAP@0.5:0.95 serving as the primary assessment criterion to comprehensively evaluate the performance of the enhanced detection model. The calculation of Equation 5 is as follows:


(5) $$mAP=\frac{\sum_{i=1}^{N}AP_{i}}{N}$$

In Equation 5, mAP denotes the value of AP calculated for all images in each category at a set IoU value, averaged over all categories.

The number of parameters was measured in megabytes (M), which quantifies the size of the model and the consumption of memory resources and is an important metric for evaluating the complexity of the model.

Giga FLOPs (GFLOPs) is a quantity that measures the computational power of a computer.

FPS refers to how many frames of image the model can process per second, which directly reflects the detection speed of the model in frames per second (frames/s). The larger the value of this indicator, the better.

### 3.3 Comparative experiments

To validate the enhanced performance of the proposed model, a comparative analysis was conducted between the constructed model and the prevailing mainstream model. The results of this comparison experiment are presented in **Table 1**. The mAP@0.5:095 of the improved YOLOv8s model in this paper was 58.87. This was the highest value and was significantly higher than the Centernet model and the Rtmdet-tiny model. The results showed that the improved WCS-YOLOv8s model was the most effective for target identification and localization during the whole strawberry growth process. The FPS of the WCS-YOLOv8s, YOLOv8s, and YOLOv6s models were all higher than 100, significantly higher than those of the other models. This indicates that the prediction speeds were faster, which met the needs of automated online detection. The model parameter numbers of the YOLOv8n and YOLOv8s models were 3.15M and 11.16M, respectively. The difference between the parameter numbers of the two models was small. The FPS values of the models were close to each other. The mAP@0.5 and mAP@0.5:0.95 values of YOLOv8s were 85.59 and 58.87, respectively. The highest mAP@0.5:0.95 value for YOLOv8s was 58.87. Considering all the factors, YOLOv8s was the best baseline model for further improvement research.

**Table 1:** Comparative experimental results of the different models.

Model	mAP@0.5 /%	mAP@0.5:095 /%	Parameter /M	FPS	Detection speed in ms per image
YOLOv8n	84.53	58.01	3.15	104.5	9.6
YOLOv8s	85.59	58.87	11.16	102.3	9.8
YOLOv8m	85.45	59.78	25.90	80.6	12.4
YOLOv8l	85.63	58.62	43.69	69.6	14.4
YOLOv8x	84.73	59.34	68.22	53.4	18.7
CenterNet	31.00	17.70	14.21	4.6	217.4
Rtmdet-tiny	79.40	46.90	4.87	9.8	102.0
Faster- rcnn	85.50	55.50	28.29	9.6	104.2
dino	85.10	58.10	47.54	9.3	107.5
YOLOv3s	82.81	54.52	103.66	59.6	16.8
YOLOv5s	83.50	55.42	9.11	91.9	10.9
YOLOv6s	82.88	54.22	16.29	101.7	9.8

### 3.4 Ablation experiments

In this paper, using YOLOv8s as the baseline model to enhance the model accuracy using Warmup data enhancement method, and fusing the use of the SE-MSDWA module and CGFM, four sets of experiments were set up to ensure the feasibility of the optimization scheme. The findings are presented in **Table 2**.

**Table 2:** Results of the ablation experiments.

Model	Warmup	SE-MSDWA	CGFM	P in %	R in %	mAP@0.5	mAP@0.5:0.95	Parameter in M	FPS	Detection speed in ms/per image
1	×	×	×	81.6	84.3	85.59	58.87	11.16	102.3	9.8
2	✓	×	×	80.3 (-1.3)	86.6 (+2.3)	86.55 (+0.96)	59.98 (+1.02)	11.16	102.3	9.8
3	×	✓	×	82.7 (+1.1)	84.5 (+0.2)	86.45 (+0.86)	59.46 (+0.59)	11.72	92.3	10.8
4	×	×	✓	81.2 (-0.4)	84.4 (+0.1)	86.47 (+0.88)	59.77 (+0.9)	18.10	48.8	20.5
5	×	✓	✓	82.5 (+0.9)	84.5 (+0.3)	86.63 (+1.04)	59.68 (+0.81)	18.69	45.9	21.8
6	✓	✓	✓	83.4 (+1.8)	86.7 (+2.4)	87.53 (+1.94)	60.48 (+1.61)	18.69	45.9	21.8

The Warmup data enhancement strategy was incorporated into the baseline model. By maintaining the original structure of the model, this strategy resulted in increases of 0.96%, 1.02%, and 2.3% in mAP@0.5, mAP@0.5:0.95, and recall, respectively. Notably, the computational effort required by the WCS-YOLOv8s model remained unchanged compared to that of the baseline model. These findings suggested that integrating the Warmup data enhancement method effectively enhances the accuracy of the model.

After incorporating the SE-MSDWA module into the backbone network of the benchmark model, there was a notable enhancement in performance metrics. Specifically, the mAP@0.5 and mAP@0.5:0.95 values of the model were improved by 0.86% and 0.59%, respectively. Additionally, the precision of the model exhibited an increase of 1.1%, indicating a significant improvement overall in its precision metrics.

After incorporating the CGFM module into the neck structure of the benchmark model, we observed an improvement in recall by 0.1%. Additionally, the precision of the new model showed a significant enhancement of 0.88% for mAP@0.5 and 0.9% for mAP@0.5:0.95 compared to the improved model; however, it is important to note that this represents a decrease of 0.4% in precision when compared to the benchmark model itself. This study focused on multi-target detection and placed greater emphasis on the mAP@0.5:0.95 metric, indicating that the integration of the CGFM module further enhanced the effectiveness of our proposed model.

As shown in **Table 1**, the YOLOv8s benchmark model comprised 11.16 M parameters and achieved a frame rate of 102.3 FPS. In contrast, the WCS-YOLOv8s improved model proposed in this paper had an increased parameter count of 18.69 M, representing an augmentation of 7.53 million parameters. The detection speed of this enhanced model was recorded at 45.9 FPS (21.8 ms per image), which sufficiently meets the requirements for automated real-time detection applications. Moreover, the precision rate, recall, mAP@0.5, and mAP@0.5:0.95 of the detection of the WCS-YOLOv8s model were 83.4%, 86.7%, 87.53%, and 60.48%, respectively. Thus, the WCS-YOLOv8s model improved on mAP@0.5, mAP@0.5:0.95, and recall by 1.94%, 1.61%, and 2.4%, respectively, and the detection accuracy rate was significantly improved. The fact that the model had the best results for each index indicated the effectiveness of the improvement. The WCS-YOLOv8s model effectively reduced the omission and misdetection of the baseline model in complex situations and improved the detection accuracy of target identification and localization during the whole process of strawberry growth.

### 3.5 Detection effect of the WCS-YOLOv8s model

**Figure 5:**
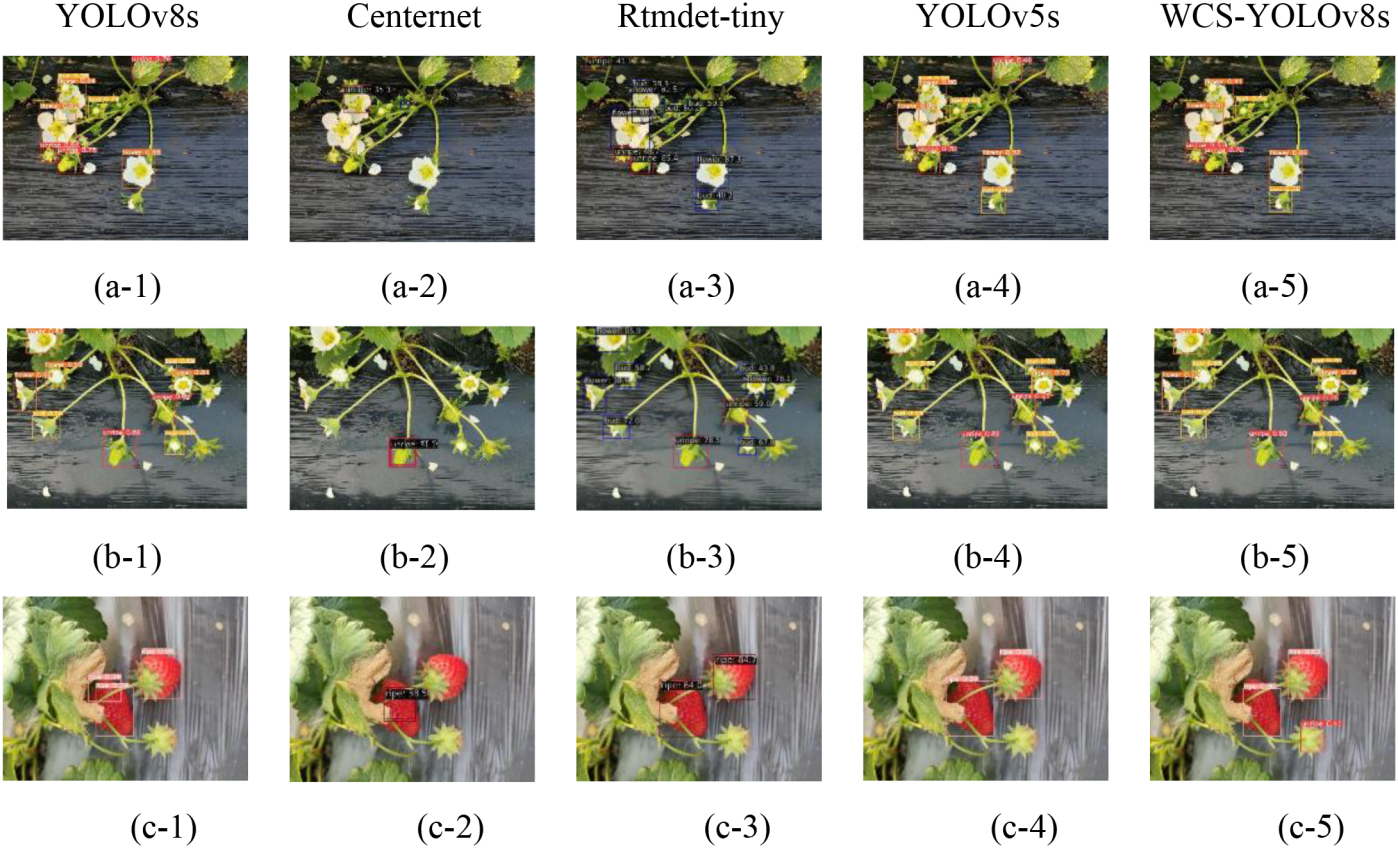
Detection results of different models under three image samples where **(a-1)**, **(b-1)**, and **(c-1)** are strawberry flower and bloom stage; strawberry bloom stage; and strawberry fruit original image samples, respectively. The image sets **(a-2, a-3, a-4, a-5)**; **(b-2, b-3, b-4, b-5)**; **(c-2, c-3, c-4, c-5)** denote the results of the recognition of the respective original image samples utilizing the YOLOv8s, Centernet, Rtmdet-tiny, YOLOv5s, and WCS-YOLOv8s models, respectively.

To enhance the evaluation of the effectiveness of the WCS-YOLOv8s model developed in this study, a comparative analysis was conducted with several current mainstream object detection models, including the YOLOv8s, CenterNet, Rtmdet-tiny, and YOLOv5s models. The results were visualized for clarity. Three images depicting strawberries in various scenes from the dataset constructed in this paper (**Figures 5a–c**) were analyzed to assess their recognition outcomes.

**Figure 5** shows the recognition results in the original benchmark YOLOv8s, CenterNet, Rtmdet-tiny, YOLOv5s, and WCS-YOLOv8s models for three scenarios. Each row of the figure shows the detection results for the same strawberry images in the YOLOv8s model, CenterNet, Rtmdet-tiny, YOLOv5s, and WCS-YOLOv8s models, respectively. From the comparison of image a-5 with a-1, a-2, a-3, and a-4, it can be seen that the YOLOv8s and YOLOv5S models misidentified a leaf in the upper right corner of the image as an immature strawberry, and the analytical reason may be that the above models were worse at detecting smaller strawberry targets. As shown in the a-2 and b-2 of **Figure 5**, the strawberry flower and bloom targets were not effectively recognized by the CenterNet model, and the detection effect was poor; from a-5 and b-5 of **Figure 5** compared with other images, the samples of four different growth periods. In the images could be detected better, and the effect was the best. As could be seen from image c-1, the YOLOv8s model detected a single ripe strawberry multiple times and incorrectly identified a single strawberry as multiple strawberries. As can be seen in c-1, c-2, c-3, and c-4, the YOLOv8s, CenterNet, Rtmdet-tiny, and YOLOv5s models failed to detect the ripe strawberries with stalks facing upwards, whereas the WCS-YOLOv8s model accurately identified them. Overall, the original YOLOv8s, CenterNet, Rtmdet-tiny, and YOLOv5s models had low accuracy when detecting small-sized strawberries and dealing with occluded targets, and were prone to omissions and false detections. Among them, the CenterNet model had the worst detection effect, with more errors and missed detections.WCS-YOLOv8s performed superiorly in small target detection, edge detection, dense detection, and branch and leaf occlusion, had significantly lower missed detections and false detections, and at the same time improved the detection confidence level.

**Figure 6:**
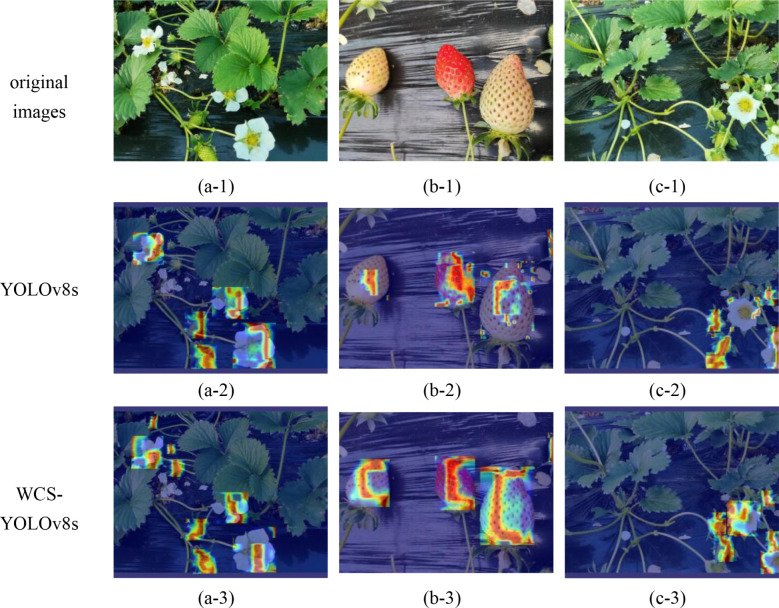
Grad-CAM heat map visualization. **(a-1)**, **(b-1)**, **(c-1)** denote the original image samples of strawberries; image sets **(a-2, a-3)**; **(b-2, b-3)**; **(c-2, c-3)** denote the respective original image samples under the YOLOv8s and wCS-YOLOv8s models, respectively, as a result of heat map visualization.

Grad-CAM (Gradient-weighted Class Activation Mapping) is a deep learning visualization technique for explaining the decision-making process of convolutional neural networks (CNNs). It made the model’s decision-making process more transparent by highlighting the image regions that the model considers most important in the image classification task, enhancing the model’s interpretability. This visualization not only helped researchers identify erroneous or irrelevant features that the model may rely on but also provided guidance for model improvement. In order to explain the WCS-YOLOv8s model’s target identification and localization of regions of interest during the whole strawberry growth process, this paper performed the Grad-CAM heat map visualization of the baseline YOLOv8s model and the improved WCS-YOLOv8s model, in which the location of the regions of interest identified by the model was visualized on the target by the blue and red zones. The Grad-CAM heat map visualization is shown in **Figure 6**.

As shown in **Figure 6**, three original images (**Figures 6a-1, b-1, c-1**) were selected for heat map visualization in this paper. The first row (a-1, b-1, and c-1) comprised the three original images; the second row (a-2, b-2, and c-2) was the heat map output from YOLOv8s; the third row (a-3, b-3, and c-3) was the heat map output from WCS-YOLOv8s. From the comparative analysis of a-3 and c-3 with a-2 and c-2 in **Figure 6**, respectively, it can be seen that the red areas cover more of the images, are more accurate, and more accurately cover small targets such as strawberry flower buds, which indicated that the area of interest focused on was more accurate when using the improved WCS-YOLOv8s for target recognition. From the comparative analysis of b-3 and c-3 with b-2 and c-2 in **Figure 6**, respectively, it can be seen that the red areas cover more area in the images and accurately cover the strawberry targets to be detected, indicating the higher accuracy of detection and the higher confidence of the category when using WCS-YOLOv8s for target recognition. It was proven by Grad-CAM heat map visualization that recognition using the WCS-YOLOv8s was better.

**Figure 7:**
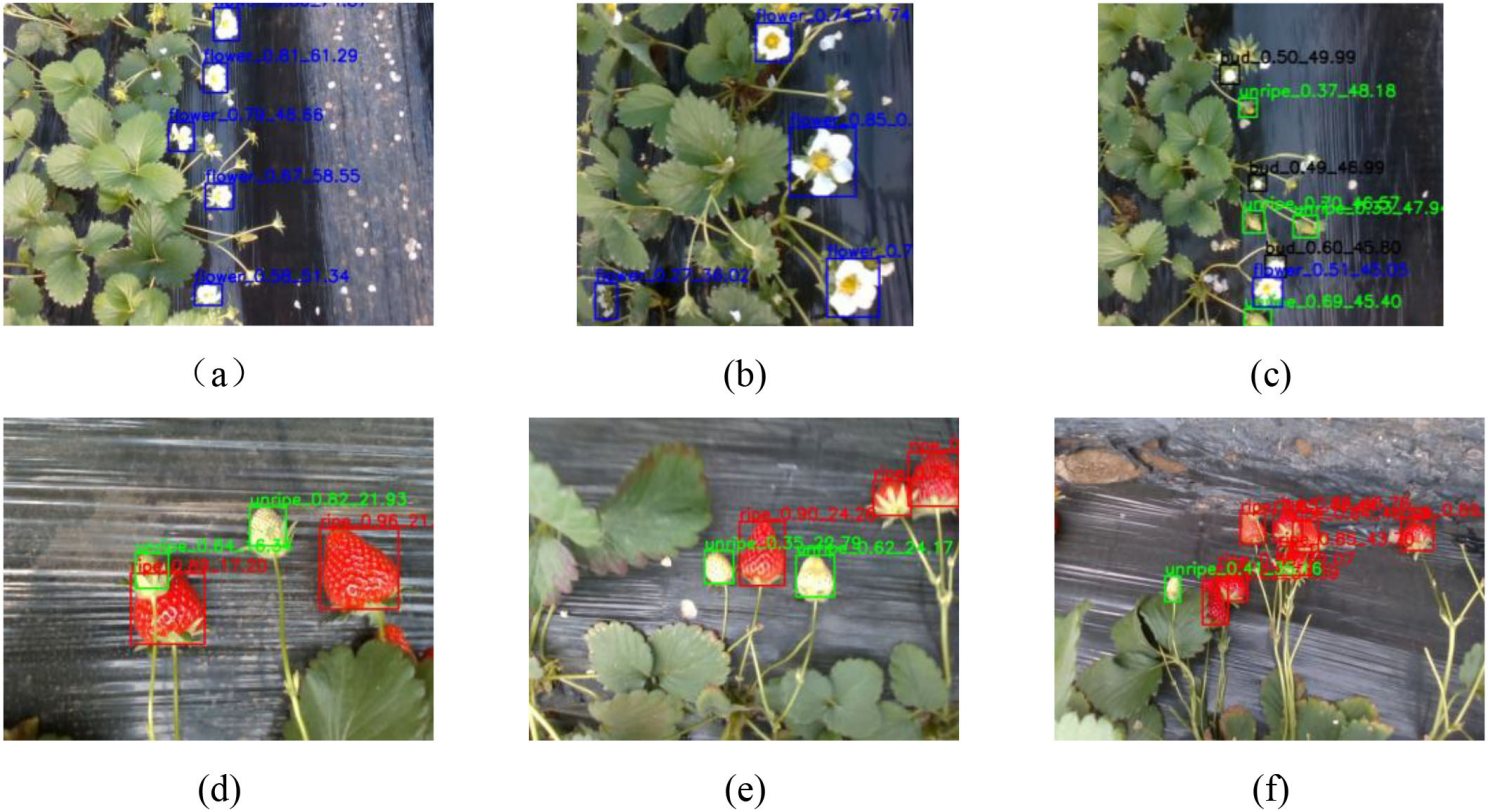
Target recognition and localization results of the improved model. The strawberry targets detected in the figure are represented by red rectangular boxes, and the markings above the rectangular boxes represent, in order, the strawberry target category, target confidence, and target detection distance from the camera. Images **(a–f)** represent the recognition results of different original images under WCSYOLOv8s Model, respectively.

In this paper, the YOLO model detected the strawberry target position in the 2D image, obtained the 2D coordinate information (x, y) of the strawberry target, and then calculated the depth information of the strawberry through the depth map, so as to obtain the x, y, and z coordinates of the strawberry target with respect to the binocular depth camera, thus realizing the accurate recognition and localization of the strawberry’s position. The results of the improved model’s target recognition and localization are shown in **Figure 7**. In **Figure 7**, the detected strawberry target is represented by a red rectangular box, and the rectangular box is labeled as follows: strawberry target category, target confidence level, and target detection distance from the camera. Four strawberries are identified in **Figure 7d**. The image contains two unripe strawberries and two ripe strawberries. Taking the unripe strawberry identified at the top of the image as an example, the probability of the improved WCS-YOLOv8s model identifying this target as an unripe strawberry was 0.82, and this unripe strawberry was 21.93 cm away from the camera. **Figures 7a, c, f** demonstrate the target detection effect of the improved algorithm in complex scenes containing multiple targets and small targets. **Figures 7b, d, e** show the detection effect of the improved algorithm in simple scenes, demonstrating that the model accurately detected targets. In summary, WCS-YOLOv8s performed well in various scenarios, proving the effectiveness of the model. The model was able to provide comprehensive intelligent target recognition and could form the basis for the realization of robotic automation to collect models that require high recognition accuracy and fast recognition speed. The model constructed in this paper can provide reliable technical support for orchard strawberry yield prediction for automated intelligent picking.

## 4 Conclusion

In order to address the issues of low efficiency, high labor intensity, time consumption, and elevated costs associated with the manual identification, localization, and supervision of strawberries, this paper presents an innovative approach by proposing an enhanced model based on the YOLOv8s framework—the WCS-YOLOv8s model. This model was employed to effectively carry out strawberry target identification and localization while facilitating comprehensive supervision throughout the entire growth process of strawberries. In this paper, the Warmup data enhancement strategy was adopted to provide a stable convergence direction at the early stage of training, which effectively avoided model oscillation and improved the robustness of the model in complex scenes. The CGFM module was introduced to fuse different information through the multi-head self-attention mechanism, which significantly improved the recognition accuracy of the model in dealing with complex scenarios, including multiple targets, small targets, and occlusion problems, and could provide a reliable method for fruit target recognition and detection in complex scenarios. The developed SE-MSDWA module effectively integrates deep separable convolution, multi-scale convolution, and the SE module. This integration enhances the capability of sample feature extraction, thereby improving both the feature extraction efficiency and overall performance of the convolutional neural network. The accuracy, recall, mAP@0.5, and mAP@0.5:0.95 of the WCS-YOLOv8s model were 83.4%, 86.7%, 87.53%, and 60.48%, respectively, with a detection speed of 45.9 FPS. When compared to the baseline YOLOv8s model, 1.94% and 1.61% improvements in mAP@0.5 and mAP@0.5:0.95 metrics were observed, respectively, thus indicating a significant enhancement in the detection accuracy of the proposed model. The WCS-YOLOv8s model established in this paper provides a reliable new method of target identification and localization for automated management and picking and quality enhancement throughout the strawberry growth process.

## Data Availability

The raw data supporting the conclusions of this article will be made available by the authors, without undue reservation.
